# Vaccination coverage with the pneumococcal and influenza vaccine among persons with chronic diseases in Shanghai, China, 2017

**DOI:** 10.1186/s12889-020-8388-3

**Published:** 2020-03-19

**Authors:** Yuheng Wang, Minna Cheng, Siyuan Wang, Fei Wu, Qinghua Yan, Qinping Yang, Yanyun Li, Xiang Guo, Chen Fu, Yan Shi, Abram L. Wagner, Matthew L. Boulton

**Affiliations:** 1 Department of Chronic Non-Communicable Diseases and Injury, Shanghai Municipal Center for Disease Control & Prevention, 1380 West Zhongshan Road, Shanghai, 200336 China; 2 National Clinical Research Center for Aging and Medicine, Huashan Hospital, Fudan University, 12 Middle Wulumuqi Road, Shanghai, 200031 China; 3 Department of Epidemiology, School of Public Health, University of Michigan, 1415 Washington Heights, Ann Arbor, 48109 MI USA; 4 Department of Internal Medicine, Division of Infectious Disease, University of Michigan Medical School, 1500 East Medical Center Drive, Ann Arbor, 48109 MI USA

**Keywords:** Vaccination, Pneumonia, Pneumococcus, Influenza, Chronic disease, China

## Abstract

**Background:**

Adults with chronic conditions such as heart disease, diabetes, or lung disease are more likely to develop complications from a number of vaccine-preventable diseases, including influenza and pneumonia. In this study, we use the data from a chronic disease management information system in Shanghai to estimate vaccination coverage and characterize predictors of seasonal influenza and 23-valent pneumococcal polysaccharide vaccine (PPSV23) vaccination among people with chronic disease in Shanghai.

**Methods:**

The Shanghai Centers for Disease Control and Prevention have information systems related to chronic disease management, hospital records, and immunizations. Data from individuals with hypertension, diabetes and chronic obstructive pulmonary disease (COPD) were abstracted during July 2017. The main outcome was coverage of pneumococcal and influenza vaccination. Vaccination coverage was calculated across demographic groups. Significance in bivariate associations was assessed through Pearson’s chi-square tests, and in multivariable models through logistic regression models with a forward stepwise method to select variables.

**Results:**

In the sample of 2,531,227 individuals ≥15 years, 22.8% were vaccinated for pneumonia from January 2013 to July 2017, and the vaccination coverage of influenza in the 2016/17 influenza season was 0.4%. Vaccination coverage was highest in those 70–79 and lowest in those younger than 60. Compared to urban areas, uptake in rural areas was higher for pneumonia vaccination (OR: 2.43, 95% CI: 2.41, 2.45), but lower for influenza vaccination (OR: 0.55, 95% CI: 0.51, 0.59). Having a greater number of chronic diseases was associated with higher likelihood of pneumonia vaccination (3 vs 1: OR: 1.68, 95% CI: 1.64, 1.71), but this relationship was not statistically significant for influenza vaccination.

**Conclusions:**

We found low levels with of pneumococcal vaccination, and extremely low uptake of influenza vaccination among individuals with high risk conditions in Shanghai who should be priority groups targeted for vaccination. Interventions could be designed to target groups with low uptake – like younger adults, and individuals who have not yet retired.

## Background

Adults with chronic conditions such as heart disease, diabetes, or lung disease are more likely to develop complications from certain vaccine-preventable diseases, especially pneumonia and influenza. These complications can include long-term illness, hospitalization, and even death [1]. Persons with diabetes or chronic obstructive pulmonary disease (COPD) often have immune system impairment sometimes leading to greater morbidity or mortality following infection with influenza compared with healthy adults of the same age. These individuals also have an influenza-related hospitalization and excess mortality rate significantly higher than those without chronic disease [2–4]. One study showed diabetics had 3.63 times higher odds of developing serious complications from the influenza compared to non-diabetics (95% confidence interval (CI): 1.15, 11.51) [5]. In one systematic review of avian influenza, people with diabetes had 9.91 times the odds of hospitalization with influenza compared to healthy people (95% CI: 5.46, 17.99) and those with COPD had 2.38 times (95% CI: 1.58, 3.57), 4.46 times (95% CI: 1.34, 14.79) and 4.02 times (95% CI: 1.69, 9.58) higher odds of hospitalization, being admitted to the ICU, and requirement ventilator assistance, respectively [6].

Several studies have found a benefit of administering pneumococcal polysaccharide and seasonal influenza vaccines to people with chronic illness [7–9]. Simultaneous vaccination of pneumococcus and influenza in elderly COPD patients could reduce pneumonia hospitalization by 63% and overall mortality by 81% [8]. Influenza vaccination could substantially reduce hospitalization and mortality among diabetic patients and was well tolerated during an influenza season [10]. The combination of seasonal influenza and pneumococcal vaccine (including 23-valent pneumococcal polysaccharide vaccine (PPSV23)) significantly reduced the hospitalization rate and mortality of influenza, pneumonia and other diseases such as respiratory disease, COPD and congestive heart failure among the elderly compared to the uptake of influenza or pneumococcal vaccine alone [11, 12]. Co-administering these vaccines could significantly reduce the rate of intensive care and prolong the survival period of elderly patients with chronic diseases [13], and has been shown to be cost-effective [14].

The elderly and patients with chronic disease including diabetes, COPD and heart disease are recommended to be priority groups for pneumococcal and influenza vaccination by the World Health Organization (WHO) [15, 16] and by the US Centers for Disease Control and Prevention (CDC) [17]. According to Chinese guidelines for vaccination, adults with these chronic diseases are recommended to receive the seasonal influenza and PPSV23 vaccines [18, 19]. Pneumococcal vaccines (PPSV23 and 13-valent pneumococcal conjugate vaccines) are also available to children for a fee. According to the manufacturer’s instructions, children and younger adults with certain chronic conditions (cardiovascular disease, lung disease, diabetes, cirrhosis, spleen dysfunction, sickle cell disease, chronic renal failure, organ transplants, HIV, cerebrospinal fluid leakage) or who live in certain environments (individuals in long-term care facilities, staff at welfare organizations) are recommended to get the PPSV23 vaccine. These recommendations are consistent with global guidelines for prevention and treatment of chronic diseases [20, 21]. The governments of some cities in China such as Beijing, Shenzhen, Karamay and Xinxiang have published policies providing free influenza vaccination to local elderly residents, while some other cities such as Chongqing and Ningbo implemented subsidies for the influenza vaccine in medical insurance programs for target residents [22]. Shanghai has implemented a government program providing people over 60 years old with a free pneumococcal vaccination (PPSV23) since 2013, but the influenza vaccine is not offered under the government’s Expanded Program on Immunization (EPI) and is instead administered for a fee.

There is a large population of chronic disease patients in Shanghai [23], but data about pneumococcal and influenza vaccination coverage among patients with chronic disease is absent. A 2010 survey from China found that influenza vaccination was actually lower in adults with high-risk health conditions (7.2%) than those without (10.8%) [24]. More information is needed about who gets vaccinated. In this study, we use the data from a chronic disease management information system in Shanghai to estimate vaccination coverage and characterize predictors of influenza and pneumococcal vaccination among people with chronic disease in Shanghai. We assess whether there are differences in coverage in pneumococcal vaccine and influenza vaccine across age groups, urbanicity and chronic disease diagnoses. We hypothesize that influenza vaccine has lower coverage than pneumococcal vaccine due to differentials in price, that uptake of both vaccines is lower in low age groups compared to high age groups, that uptake of both vaccines is lower in rural areas than in urban areas, and that coverage for both vaccines is higher among those with more chronic diseases.

## Methods

### Data sources

This study used a retrospective cohort design. During July 2017, the data were obtained from three distinct sources – (1) the Shanghai Chronic Disease Management Information System and (2) the Shanghai Immunization Program Information System which are both housed at the Shanghai CDC, and (3) the hospital record system, which is located at the Shanghai Health Commission for hospital records. The individual’s personal ID was used to link the three information systems.

Throughout Shanghai, patients ≥15 years old diagnosed with hypertension and diabetes are asked if they want to be included in a centralized database – the Chronic Disease Management Information System. Inclusion in the database means that the patients will receive more standardized management of their disease. An estimated 50% of individuals with hypertension and diabetes in Shanghai are enrolled in this database. The other 50% include those who do not know they have a chronic disease, who have not gone to visit the doctor, or who are unwilling to be enrolled into the system. General practitioners follow up with patients every 3 months at community health centers and input data related to these visits into the Chronic Disease Management Information System. This database contains information on sex, birthdate, township residence, occupation and diagnostic information pertaining to hypertension and diabetes. No other individual-level information was available from the dataset. All patients from the Shanghai Chronic Disease Management Information System were included in this study.

Data in the Immunization Program Information System were captured and entered by vaccination providers at community health care centers. Data are uploaded daily from these health centers’ electronic registries into the Immunization Program Information System. Pneumococcal vaccination information from January 2013 to July 2017 and influenza vaccination information from the 2016/17 influenza season were obtained from the Shanghai Immunization Program Information System. Types and dates of vaccination were extracted from the Immunization Program Information System. The Shanghai CDC and the Shanghai Health Commission implement regular data quality checks of the Immunization Program Information System.

Diagnosis of COPD was obtained from the hospital record system. The International Classification of Diseases (ICD) was used to define chronic diseases in the Chronic Disease Management Information System and the hospital record system. Hypertension was defined as I10-I13, diabetes was defined as E10-E14 and COPD was defined as J44.

The chronic diseases in this study represent those at risk for pneumococcal disease or influenza [15, 25]. The American Diabetes Association recommends individuals with diabetes to have both vaccines [26]. The Global Initiative for Chronic Obstructive Lung Disease has similar recommendations for those with COPD [27]. Hypertension is not thought to be linked to either disease, but individuals with hypertension were still included because they were in the original Chronic Disease Management Information System and because many are older, and thus may be age-eligible for a free PPSV23 in Shanghai.

Urbanicity was defined by characteristics of the township where participants resided. Residency status refers to locals vs. non-locals, with locals defined as registered permanent residents of Shanghai, and non-locals as migrants from other cities who have moved into Shanghai for over 6 months. Urban areas are those where ≤30% of locals and ≤ 35% of non-locals were engaged in agricultural work; suburban areas had ≤30% of locals but > 35% of non-locals engaged in agricultural work; and rural areas had > 30% of locals in agricultural occupations.

Classification of occupation was defined according to the China National Standard [28].

### Statistical analysis

The main outcome was receipt of pneumococcal and influenza vaccination. Vaccination coverage was calculated by sex, age group, urbanicity, occupation, type and number of chronic diseases. Pearson’s chi-square test was used to compare the vaccination coverage among the different subgroups. We also analyzed the relationship between predictor variables (sex, age group, urbanicity, occupation, type and number of chronic diseases) and the outcomes using logistic regression models through a forward stepwise method (variable included at *p*-value of 0.05, excluded at p-value of 0.10, with α = 0.05). Data were analyzed using SPSS version 20.

Vaccination status by township was mapped with QGIS 3.6 (QGIS Geographic Information System. Open Source Geospatial Foundation Project). The shapefile map was obtained from Shanghai Surveying and Mapping Institute (https://www.shsmi.cn/info/iList.jsp?cat_id=10098).

## Results

The sample of 2,531,227 patients from the chronic disease management information system included a majority of females (53.7%), more individuals above 60 years (78.7%) than other age groups, more urban residents than other locales (59.4%), and most individuals were retired (82.6%). The majority of patients had hypertension (90.3%) with fewer diagnosed with diabetes (31.0%) and COPD (10.3%); a very low proportion had been diagnosed with all three (3.2%) (Table 1).

**Table 1:** Demographic characteristics and vaccination outcomes of patients with chronic disease, Shanghai, July 2017

	N (%)	Pneumococcal vaccination				Influenza vaccination		
		Vaccinated	Not vaccinated	*P*-value		Vaccinated	Not vaccinated	*P*-value
Total	2,531,227	578,203 (22.8%)	1,953,024 (77.2%)			9227 (0.4%)	2,522,000 (99.6%)	
Sex								
Male	1,171,879 (46.3%)	267,988 (22.9%)	903,891 (77.1%)	0.371		4042 (0.3%)	1,167,837 (99.7%)	< 0.001
Female	1,359,348 (53.7%)	310,215 (22.8%)	1,049,133 (77.2%)			5185 (0.4%)	1,354,163 (99.6%)	
Age (years)								
15–39	25,541 (1.0%)	4 (0.0%)	25,537 (100.0%)	< 0.001		28 (0.1%)	25,513 (99.9%)	< 0.001
40–49	88,958 (3.5%)	33 (0.0%)	88,925 (100.0%)			89 (0.1%)	88,869 (99.9%)	
50–59	424,266 (16.8%)	5185 (1.2%)	419,081 (98.8%)			506 (0.1%)	423,760 (99.9%)	
60–69	925,951 (36.6%)	262,163 (28.3%)	663,788 (71.7%)			3025 (0.3%)	922,926 (99.7%)	
70–79	593,124 (23.4%)	211,679 (35.7%)	381,445 (64.3%)			3576 (0.6%)	589,548 (99.4%)	
≥ 80	473,387 (18.7%)	99,139 (20.9%)	374,248 (79.1%)			2003 (0.4%)	471,384 (99.6%)	
Area								
Urban	1,504,176 (59.4%)	281,808 (18.7%)	1,222,368 (81.3%)	< 0.001		6649 (0.4%)	1,497,527 (99.6%)	< 0.001
Suburban	482,883 (19.1%)	131,610 (27.3%)	351,273 (72.7%)			1590 (0.3%)	481,293 (99.7%)	
Rural	544,168 (21.5%)	164,785 (30.3%)	379,383 (69.7%)			988 (0.2%)	543,180 (99.8%)	
Occupation								
Government department and public institution	10,315 (0.4%)	1842 (17.9%)	8473 (82.1%)	< 0.001		44 (0.4%)	10,271 (99.6%)	< 0.001
Professional	51,195 (2.0%)	9264 (18.1%)	41,931 (81.9%)			305 (0.6%)	50,890 (99.4%)	
Clerk	45,582 (1.8%)	7761 (17.0%)	37,821 (83.0%)			187 (0.4%)	45,395 (99.6%)	
Business and service	33,708 (1.3%)	4206 (12.5%)	29,502 (87.5%)			115 (0.3%)	33,593 (99.7%)	
Farmer	168,332 (6.7%)	48,012 (28.5%)	120,320 (71.5%)			199 (0.1%)	168,133 (99.9%)	
Production and transportation	74,127 (2.9%)	11,267 (15.2%)	62,860 (84.8%)			216 (0.3%)	73,911 (99.7%)	
Army	3341 (0.1%)	1015 (30.4%)	2326 (69.6%)			6 (0.2%)	3335 (99.8%)	
Retired	2,091,689 (82.6%)	494,821 (23.7%)	1,596,868 (76.3%)			8112 (0.4%)	2,083,577 (99.6%)	
Other	52,938 (2.1%)	15 (0.0%)	51,923 (100.0%)			43 (0.1%)	52,895 (99.9%)	
Variety of chronic disease								
Hypertension	2,285,586 (90.3%)	536,089 (23.5%)	1,749,497 (76.5%)	< 0.001		8577 (0.4%)	2,277,009 (99.6%)	< 0.001
Diabetes	783,648 (31.0%)	188,896 (24.1%)	594,752 (75.9%)			3092 (0.4%)	780,556 (99.6%)	
COPD	260,012 (10.3%)	79,031 (30.4%)	180,981 (69.6%)			2213 (0.9%)	257,799 (99.1%)	
Number of chronic diseases								
1	1,812,907 (71.6%)	378,172 (20.9%)	1,434,735 (79.1%)	< 0.001		5254 (0.3%)	1,807,653 (99.7%)	< 0.001
2	638,621 (25.2%)	174,249 (27.3%)	464,372 (72.7%)			3291 (0.5%)	635,330 (99.5%)	
3	79,699 (3.2%)	578,203 (36.3%)	1,953,024 (63.7%)			682 (0.9%)	79,017 (99.1%)	
Pneumococcal vaccination								
Vaccinated						7360 (1.3%)	570,843 (98.7%)	< 0.001
Not vaccinated						1867 (0.1%)	1,951,157 (99.9%)	

Only 22.8% patients were vaccinated for pneumococcal from January 2013 to July 2017, and vaccination coverage of influenza in 2016/17 influenza season was exceedingly low at 0.4%. Vaccination coverage differed significantly across most socio-demographic characteristics. For both pneumonia and influenza vaccinations, coverage was highest in those 70–79 years (35.7 and 0.6%, respectively) compared to other age groups (*p* < 0.001). Pneumococcal vaccination was highest in rural areas (30.3% compared to 18.7% in urban areas, *p* < 0.001) whereas influenza vaccination was highest in urban areas (0.4% compared to 0.2% in rural areas, *p* < 0.001). For both pneumococcal and influenza vaccination, coverage was highest among those with COPD (30.4 and 0.9%, respectively), compared to those with hypertension (23.5 and 0.4%, respectively) or diabetes (24.1 and 0.4%, respectively) (*p* < 0.001, respectively). There was a dose-response relationship between number of chronic diseases and vaccination coverage; pneumococcal vaccination uptake was 36.3% among those with three conditions, compared to 27.3 and 20.9% for those with 2 or only 1 condition (*p* < 0.001). Influenza vaccination coverage was 0.3, 0.5 and 0.9% for those with 1, 2, or 3 conditions (*p* < 0.001).

**Fig. 1:**
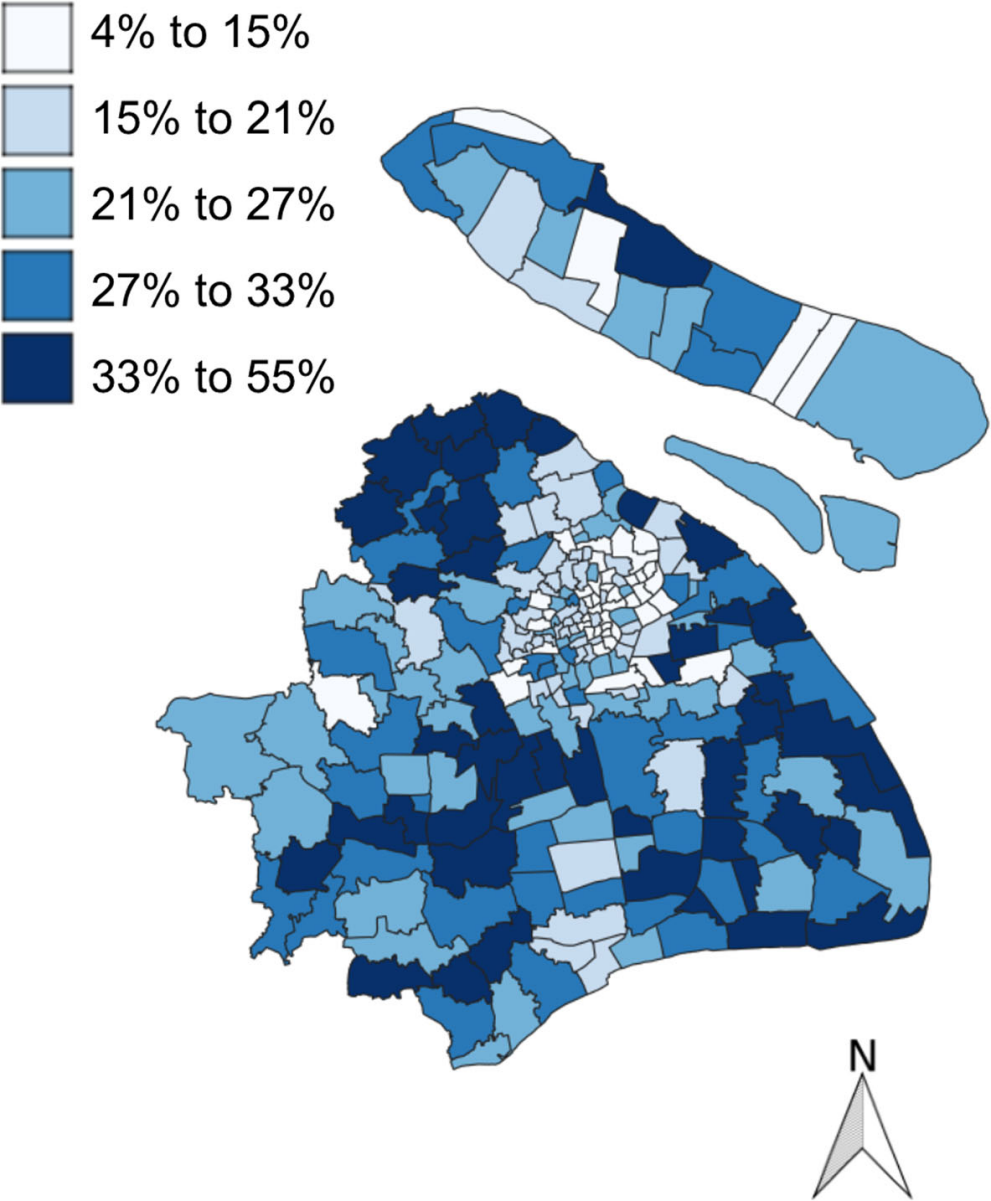
Pneumococcal vaccination coverage by township in Shanghai. The map was from Shanghai Surveying and Mapping Institute and with permission to publish

Vaccination coverage also varied geographically, with pneumococcal vaccination coverage highest in Jiading and Songjiang, at the periphery of Shanghai, and was relatively low in the inner districts of Huangpu, Jing’an, Hongkou, and Yangpu (Fig. 1). Influenza vaccination coverage was comparatively low across all districts, ranging from 0.1% in Fengxian to 0.8% in Xuhui.

Table 2 shows the multivariable logistic regression models. These models are largely in line with the unadjusted results from Table 1. Individuals aged 70–79 had 1.37 times higher odds of pneumococcal vaccine uptake compared to individuals in their 60s (95% CI: 1.36, 1.38). Individuals in rural areas and suburban area had higher odds of pneumococcal vaccine uptake compared to individuals in urban areas. Patients with 2 and 3 chronic diseases had respectively 1.31 (95% CI: 1.30, 1.33) and 1.68 (95% CI: 1.64, 1.71) times higher odds of vaccination compared to patients with 1 chronic disease.

**Table 2:** Odds ratios (OR) and 95% confidence intervals (CI) for pneumococcal and influenza vaccination among patients with chronic disease, Shanghai, July 2017

	Pneumococcal vaccination			Influenza vaccination	
	Unadjusted model OR (95% CI)	Adjusted model OR (95% CI)		Unadjusted model OR (95% CI)	Adjusted model OR (95% CI)
Sex (vs male)	1.00 (0.99, 1.00)	0.95 (0.94, 0.96) ^*^		1.11 (1.06, 1.15) ^*^	1.05 (1.01, 1.10) ^*^
Age (years) (vs 60–69)					
15–39	0.00 (0.00, 0.00) ^*^	0.00 (0.00, 0.00) ^*^		0.34 (0.23, 0.49) ^*^	0.44 (0.30, 0.66) ^*^
40–49	0.00 (0.00, 0.00) ^*^	0.00 (0.00, 0.00) ^*^		0.31 (0.25, 0.38) ^*^	0.43 (0.33, 0.55) ^*^
50–59	0.03 (0.03, 0.03) ^*^	0.03 (0.03, 0.03) ^*^		0.36 (0.33, 0.40) ^*^	0.42 (0.38, 0.46) ^*^
70–79	1.41 (1.40, 1.42) ^*^	1.37 (1.36, 1.38) ^*^		1.85 (1.76, 1.94) ^*^	1.73 (1.64, 1.81) ^*^
≥ 80	0.67 (0.67, 0.68) ^*^	0.67 (0.66, 0.68) ^*^		1.30 (1.23, 1.37) ^*^	1.09 (1.01, 1.16) ^*^
Area (vs urban)					
Suburban	1.63 (1.61, 1.64) ^*^	1.94 (1.92, 1.95) ^*^		0.74 (0.70, 0.79) ^*^	0.86 (0.82, 0.91) ^*^
Rural	1.88 (1.87, 1.90) ^*^	2.43 (2.41, 2.45) ^*^		0.41 (0.38, 0.44) ^*^	0.55 (0.51, 0.59) ^*^
Occupation (vs retired)					
Government department and public institution	0.70 (0.67, 0.74) ^*^	0.99 (0.94, 1.05)		1.10 (0.82, 1.48)	1.28 (0.95, 1.72)
Professional	0.71 (0.70, 0.73) ^*^	0.95 (0.92, 0.97) ^*^		1.54 (1.37, 1.73) ^*^	1.53 (1.37, 1.72) ^*^
Clerk	0.66 (0.65, 0.68) ^*^	0.97 (0.94, 0.99) ^*^		1.06 (0.92, 1.22)	1.08 (0.93, 1.25)
Business and service	0.46 (0.45, 0.48) ^*^	0.73 (0.70, 0.75) ^*^		0.88 (0.73, 1.06)	1.01 (0.84, 1.21)
Farmer	1.29 (1.27, 1.30) ^*^	0.93 (0.91, 0.94) ^*^		0.30 (0.26, 0.35) ^*^	0.49 (0.43, 0.57) ^*^
Production and transportation	0.58 (0.57, 0.59) ^*^	0.79 (0.77, 0.80) ^*^		0.75 (0.66, 0.86) ^*^	0.82 (0.72, 0.94) ^*^
Army	1.41 (1.31,1.52) ^*^	0.81 (0.75, 0.87) ^*^		0.46 (0.21, 1.03)	0.51 (0.23, 1.13)
Other	0.00 (0.00, 0.00) ^*^	0.75 (0.39, 1.44)		0.21 (0.16, 0.28) ^*^	0.68 (0.46, 0.99) ^*^
Hypertension (vs no)	1.48 (1.47, 1.50) ^*^	1.18 (1.16, 1.20) ^*^		1.42 (1.31, 1.54) ^*^	1.21 (1.12, 1.31) ^*^
Diabetes (vs no)	1.11 (1.10, 1.12) ^*^	0.90 (0.89, 0.92) ^*^		1.12 (1.08, 1.17) ^*^	
COPD (vs no)	1.55 (1.54, 1.56) ^*^			2.77 (2.64, 2.91) ^*^	1.91 (1.78, 2.04) ^*^
Number of chronic diseases (vs 1)					
2	1.42 (1.41, 1.43) ^*^	1.31 (1.30, 1.33) ^*^		1.78 (1.71, 1.86) ^*^	1.14 (1.08, 1.21) ^*^
3	1.81 (1.79,1.84) ^*^	1.68 (1.64, 1.71) ^*^		2.97 (2.74, 3.22) ^*^	1.08 (0.97, 1.20)
Pneumococcal vaccination (vs no)				13.47 (12.81, 14.18) ^*^	15.75 (14.86, 16.68) ^*^

^*^p < 0.05

All subjects were included in the multivariable analysis. In the adjusted model of influenza vaccination, patients aged 70–79 and above 80 had 1.73 (95% CI: 1.64, 1.81) and 1.09 (95% CI: 1.01, 1.16) times higher odds of vaccination, respectively, compared to patients aged 60–69. Compared to patients in urban area, patients in suburban and rural areas had, respectively, 0.86 (95% CI: 0.82, 0.91) and 0.55 (95% CI: 0.51, 0.59) times the odds of influenza vaccination. Patients with 2 chronic diseases had 1.14 times the odds of uptake influenza vaccine compared to patients with 1 kind of chronic disease (95% CI: 1.08, 1.21), but there was no significant difference in those with 1 vs 3 chronic diseases. Patients with a dose of pneumococcal vaccine had 15.75 the odds of receiving the influenza vaccine compared to those with no pneumococcal vaccine (95% CI: 14.86, 16.68).

## Discussion

Influenza and pneumococcal vaccination are important for preventing illness and the elderly with chronic diseases [7–9]. In a large sample of individuals with chronic diseases residing in Shanghai, China, we found low pneumococcal vaccination coverage over a 4-year study period and even lower influenza vaccine coverage. Uptake of both vaccines increased in those with more chronic diseases and with older age.

Chronic disease patients should be targeted for attaining high vaccination coverage compared to the remaining population. There are several overriding factors for exceptionally low coverage of pneumococcal and influenza vaccination among chronic disease patients in Shanghai community: (1) Studies have found that individuals lack awareness of pneumococcal and influenza vaccine [29, 30], and physicians do not often recommend vaccinations. (2) Vaccination for adults is not convenient. Community health care centers were responsible for implementing vaccinations in Shanghai. Most centers only provide 1 or 2 half days available for adult vaccination per week, while 6 half days are available for childhood vaccination. (3) Some adverse news related to vaccines have made people reduce their trust in vaccination programs [31, 32]. People with chronic disease and the elderly should have priority to take these vaccines due to their risk factors, but their chronic diseases may lead them to believe they have a higher risk for adverse reactions. (4) There is a limited supply of influenza vaccine. These reasons were not assessed in the current study, but could be explored in future research.

Pneumococcal vaccination coverage among adults 19–64 years at increased risk for pneumococcal disease was 24.0% in 2016 in the United States although it was much higher at 66.9% among adults over 65 years old [33]. This is consistent with a study from Spain showing a higher proportion of adults over 65 years had received the pneumococcal vaccine (43.8%) [34] and demonstrating that vaccination levels in both young and elderly chronic disease patients in Shanghai are substantially lower than those found in the US or Spain. Because residents over 60 years of age in Shanghai are provided with free pneumococcal vaccination, the coverage in this age groups was not surprisingly higher than younger age groups and approaching that seen in those over 65 years in Hong Kong in 2015 (34%) [35] which also offers free pneumococcal vaccination to the elderly [36].

In our study, less than 1% of individuals received an influenza vaccine, which is far lower than in other countries, many of which provide free vaccine through government-sponsored or private insurance programs. Similar studies have shown higher influenza vaccination coverage in the United States (43.5%, among adults over 19 years, 2015/16 season) [33], UK (56.0%, chronic disease patients, 2007/08 season) [37], Poland (11.1%, chronic disease patients, 2007/08 season) [37], Korea (45.2%, over 40 years, 2012) [38], and Hong Kong (39%, over 65 years, 2015) [35]. Our findings were relatively consistent with prior studies in China showing an average national vaccination coverage ranging between 1.5 and 2.2% in 2004 and 2014 [22]. The coverage among patients over 60 years was significantly higher vs younger age groups below which was almost non-existent (i.e. close to 0%). One previous study found that elderly individuals who live with other family members are more likely to get vaccinated [30], perhaps as a result of other family members thinking the elderly, but not younger adults, need to get vaccinated or elderly individuals wanting to protect themselves against influenza as they care for their grandchildren.

We found that pneumococcal vaccination coverage was higher in rural areas which distinctly contrasted with influenza vaccination coverage which was lowest in rural areas. For influenza vaccination – which requires payment, individuals in urban area might be more able to afford the cost of influenza vaccine while patients in rural area might not [39]. Higher pneumococcal vaccination coverage in rural areas may result from individuals trusting health care workers more [40].

The study showed that patients with multiple chronic diseases would be more likely to take pneumococcal vaccination than those with only one kind of chronic disease. This association could arise for several reasons. Individuals may perceive a greater personal risk of disease as they gain experience with more diseases. Or individuals with more co-morbid chronic diseases may have had more opportunities to get immunized through having more healthcare encounters.

The overall difference in uptake between influenza and pneumococcal vaccination could also be tied back to experiences and risk perceptions, as influenza could be seen as a nuisance disease that will quickly pass [41]. The lack of funding to influenza vaccination from the government might be another important reason.

Pneumococcal vaccine uptake was a strong predictor of influenza vaccine uptake, which indicates that acceptance of one vaccine probably predicts for acceptable of others. Since the observation of pneumococcal vaccination was from January 2013 to July 2017 and the observation of influenza vaccination was only 2016/17 season, the impact of influenza vaccination on pneumonia vaccination could not be calculated for temporal reasons. Co-administration of both pneumococcal and influenza vaccines could reduce the incidence of various complications, hospitalization and mortality of chronic disease [8, 11, 12]. Only 0.3% of total sample had taken both pneumococcal and influenza vaccine in 2016/17 season, lower than that of hospitalized persons aged over 65 years in Victoria (46.6%) [42].

Our study looked at vaccination coverage for influenza and pneumococcal disease including predictors for vaccination among community members in Shanghai with chronic diseases. Interventions or policies like government funding as a potential strategy to encourage vaccination, especially influenza vaccination among chronic disease patients, should be implemented. Future studies should further examine differences in uptake of vaccines across different demographic groups.

### Strengths and limitations

There are several strengths and limitations to this study. A strength of this study is the use of several comprehensive information systems as data sources, and the large number of individuals in the chronic disease management system. This system is opt-in for individuals with certain chronic diseases in the municipality, and an estimated 50% of individuals with chronic disease participate in it. It is possible that the individuals who participate in the chronic disease management system differ from those who do not. Non-participants, for example, likely have lower health-seeking behaviors and so our estimates of vaccination coverage may overestimate trends in the entire population of those with these chronic diseases. Future studies could evaluate why and how individuals participate in this database. In addition, limitations include a lack of information on key variables, like education and income. We only have data of pneumococcal vaccination coverage from 2013 onward and 1 season of influenza vaccination coverage, and inclusion of additional years would have permitted analysis of trends over time. The very low vaccination coverage, particularly for influenza vaccination, limits our ability to make recommendations beyond a general recommendation to increase coverage.

## Conclusions

We found very low levels of both pneumococcal and influenza vaccination among individuals with chronic diseases residing in Shanghai. These individuals should be prioritized for vaccination with both vaccines. Concomitantly, there can be greater ease of access to vaccines, and promotional materials can focus on complications of disease in those with high risk conditions.

## Data Availability

The datasets analyzed for the current study are not publicly available because they contain detailed medical histories of chronic patients, but are available from the corresponding author on reasonable request.

## Abbreviations

CDC: Centers for Disease Control and Prevention
CI: Confidence interval
COPD: chronic obstructive pulmonary disease
EPI: Expanded Program on Immunization
ICD: International Classification of Diseases
OR: Odds ratio
PPSV23: 23-valent pneumococcal polysaccharide vaccine
